# Disparities in patient-resident physician communication and counseling: A multi-perspective exploratory qualitative study

**DOI:** 10.1371/journal.pone.0288549

**Published:** 2023-10-23

**Authors:** Asma Altaf Hussain Merchant, Namra Qadeer Shaikh, Noreen Afzal, Ali Aahil Noorali, Komal Abdul Rahim, Rida Ahmad, Areesha Ahmer, Adnan Ali Khan, Saqib Kamran Bakhshi, Saad bin Zafar Mahmood, Maryam Pyar Ali Lakhdir, Muhammad Rizwan Khan, Muhammad Tariq, Adil H. Haider

**Affiliations:** 1 Dean’s Office, Aga Khan University, Karachi, Pakistan; 2 Department of Medicine, Aga Khan University, Karachi, Pakistan; 3 Department of Community Health Sciences, Aga Khan University, Karachi, Pakistan; 4 Medical College, Aga Khan University, Karachi, Pakistan; 5 Department of Surgery, Neurosurgery, Aga Khan University, Karachi, Pakistan; 6 Department of Surgery, Aga Khan University, Karachi, Pakistan; University of Sharjah College of Health Sciences, UNITED ARAB EMIRATES

## Abstract

Effective communication between physicians and patients plays an integral role in clinical care. Gaps in a physician’s ability to ensure effective communication, especially with patients from diverse backgrounds, are known causes of medical errors. This study explores the potential biases and disparities in patient-resident communication, which may influence a patient’s quality of care. This exploratory qualitative study was conducted at the largest academic medical center in Pakistan. Purposive sampling was used to approach participants from surgery, medicine, obstetrics and gynecology, pediatrics and family medicine. Faculty, fellows and residents working in these departments and medical students in their fourth and fifth years of undergraduate education with prior experience of at least one month in these specialties during their clinical rotations were included. Focus group discussions (FGDs) lasting 45–60 minutes were conducted with each cohort of healthcare professionals separately, using a semi-structured interview guide. Sixty participants (19 males and 41 females, mean age: 32.9, SD: 10.9) took part in the study. Thematic analysis revealed five major themes. Four themes focused on residents’ biases and patient disparities hindering patient-resident communication: (1) patient-resident gender discordance (2) ethnicity and language barriers, (3) differing social class of the patient, and (4) challenging patient-resident interactions (patients resistant to treatment, exceedingly inquisitive and those with multiple attendants, etc.). The fifth theme identified the need for a communication skills curriculum in postgraduate medical education. Opposite gender and discordant socioeconomic/cultural backgrounds of patients pose a challenge to effective patient-physician communication. Self-identification and awareness of residents’ biases when interacting with patients can ensure their active elimination and improve their communication skills. Integrating these components in a standardized curriculum within postgraduate programs can enable resident-physicians to provide the same level of care and communicate more efficiently with patients of all backgrounds.

## Introduction

Effective communication lies at the core of providing quality care to patients [1]. Developing strong communication with patients aids doctors in building therapeutic relationships, facilitating exchange of information pertinent to the patient’s health, and taking into account the patients’ decisions in their plan of care [2]. It is vital for physicians, especially residents, to obtain a comprehensive patient history to make an accurate diagnosis and provide appropriate care.

Whilst communication skills are of utmost importance, interpersonal skills also play a fundamental role in establishing a trustworthy and respectful patient-doctor relationship [3]. Incorporating empathy while developing therapeutic relationships, regardless of patients’ backgrounds, assists physicians in implementing a patient-centered approach from the initial stages of treatment. Hence, the quality of patient care and outcomes improve with an appropriate ‘bedside manner’—often considered reflective of a doctor’s competency by patients [1]. However, difficult patient encounters, demanding families and differences of opinions on treatment plans can frustrate physicians, especially inexperienced residents, negatively impacting the patient-doctor relationship [4].

Even though equality in patient care is essential, literature demonstrates poor-quality communication by clinicians if patients belong to racial/ethnic minorities or have a low socioeconomic background [3, 5]. This is often associated with unconscious preferences, otherwise known as implicit bias, exhibited by physicians especially when there is a dyad with a differing socioeconomic status (SES). This may manifest in physicians’ non-verbal communication and decision-making [5], resulting in sub-optimal outcomes for an already vulnerable population [6]. Resident-physicians are more susceptible to show implicit bias since they are inadequately prepared to provide cross-cultural care [7]. Studies using the Implicit Association Test (IAT) indicate presence of unconscious race and social class bias towards patients among residents, fellows and faculty, with a strong preference for upper social class patients [8].

Literature from the United States has explored the impact of racial and ethnic biases on patient-provider communication, where the latter includes both residents and physicians [9]. This affects the dialogue poorly, leading to poor adherence of medical advice by patients of color. Multiple modules integrated within the residency program, such as low-cost, high-impact anti-bias modules and ask-tell-ask model of communication have shown to improve racial consideration among residents when conversing with their patients [7, 10]. However, the impact of biases pertaining to gender and socioeconomic status still needs to be understood.

While this subject is gaining recognition in the West, there is a dearth of literature in low-middle income countries (LMICs) on residents’ biases towards patients. The limited number of studies investigating this domain reflects patient dissatisfaction with the communication and interpersonal skills exhibited by physicians [11]. As a means to understand this aspect in LMICs, this study aimed to explore potential biases and disparities amongst resident-physicians while interacting with patients. Identification of these biases amongst resident-physicians can lay the foundation for policy makers to devise relevant and contextual solutions that can be implemented during postgraduate training to improve patient-resident communication.

## Methodology

### Study design

The consolidated criteria for reporting qualitative research (COREQ) checklist were used to design this study (S1 File) [12]. Using an exploratory descriptive qualitative study design, purposive sampling was used to approach participants [13]. Data was collected over three months (July 2021 –September 2021). A detailed description of the methodology is described in another paper [14].

### Setting and participants

This study was conducted at the Aga Khan University Hospital (AKUH), the largest Academic Medical Center (AMC) in Pakistan. Accredited by the Joint Commission International (JCI), this 650-bed hospital offers residency programs recognized by the Accreditation Council for Graduate Medical Education (ACGME).

### Eligibility criteria for participant selection

Faculty, fellows and resident-physicians working in the departments of surgery, medicine, obstetrics and gynecology, pediatrics, and family medicine for at least three months were eligible to take part in the study. Fourth- and fifth-year medical students having prior experience of at least one month in these specialties during their clinical rotations were included.

Faculty members working as clinical lecturers and/or part-time physicians and medical students who may have completed 3 years of medical education but not completed their rotation with the specified departments were not eligible to take part in the study.

### Data collection

A semi-structured interview guide was developed through an extensive literature search and review by the team’s qualitative experts. Open-ended questions with probes centered around participants’ observations of patient-resident communication, factors hindering empathetic communication, possible biases of residents towards patients and recommendations for improving patient-resident communication. Using this guide, focus group discussions (FGDs) were conducted with faculty, fellows, residents, and medical students.

Participants were approached via their institutional email addresses to provide the study’s background and sign-up forms for voluntary participation in FGDs. Those who signed up were then invited to choose a convenient time for the discussion. Six research team members (AAHM, AAK, AAN, NA, NQ and RA) conducted the FGDs. Training was provided to them to ensure standardization of data collection. The interviewers comprised of both male and female members of the research team. AAN, NQ, RA (Bachelor of Medicine, Bachelor of Surgery) and NA (M.Phil. Clinical Psychology) were research associates while AAK and AAHM were final year medical students. FGDs were conducted within the confines of enclosed conference rooms in the hospital premises. A prior relationship with participants was not established and only the research team members and participants were present during the FGDs. Each cohort of health care providers (HCPs) were interviewed once. The FGDs lasted for 45–60 minutes each and were audio recorded. Notes were also taken during the FGDs to capture additional details. Data collection continued until thematic saturation was reached and repeat FGDs were not conducted.

### Data analysis

Audio recordings of the FGDs were transcribed verbatim. Transcripts were then uploaded on NVivo version 11 (QSR International). Braun and Clark’s six-step method for thematic analysis was followed for data analysis [15]. The first step requires researchers to familiarize themselves with the data. Since the research staff carrying out the analysis (NA and AAHM) also performed transcription, this helped them become immersed in the data. They also read each transcript multiple times for familiarization with the data. In the second step, the researchers inductively and independently coded transcripts line by line, to generate an initial list of codes. This was followed by a discussion to decide the final list of codes to be applied to all transcripts. The next step was searching for themes in the data. All codes were reviewed to identify areas of similarity. To highlight patterns in the data, similar codes were grouped to form subthemes. Independent themes were then constructed using all codes falling under each theme. The fourth step involved reviewing and comparing the themes against the data to identify any more potential themes or to revise existing ones. This step was then followed by clear naming of each theme to adequately describe its content. As a last step, all themes were organized in a way for them to build up on prior themes so that data could be described in a logical manner. Participant quotations corresponding to each theme were paired with their relevant text.

### Rigor

Based on Lincoln and Guba’s criteria for establishing trustworthiness of the findings [16], triangulation of the investigators (two data coders) and data sources (investigating the viewpoints of four different groups of respondents) were used to establish credibility of the findings. The researchers also made reflexive notes during data collection to be mindful of their own preconceived ideas about patient-physician communication.

### Ethical considerations

This study received approval from the Ethics Review Committee at the Aga Khan University (2021-6041-17126). Participants signed a written informed consent form before participating in the FGDs which conveyed relevant study details.

### Results

Eight FGDs were conducted with sixty participants, details of which are provided in Table 1. These included two FGDs each with faculty, fellows, residents, and medical students.

**Table 1:** Characteristics of FGD study participants (n = 60).

Characteristics of participants		Total (%)	Gender	
			Male (%)	Female (%)
**Designation **	Medical Students	18 (30)	7 (39)	11 (61)
	Fellows	12 (20)	5 (42)	7 (58)
	Residents	14 (23)	3 (21)	11 (79)
	Faculty	16 (27)	4 (25)	12 (75)

Thematic analysis revealed four major themes describing biases and disparities in patient-resident communication, alongside a fifth theme recommending a communication skills curriculum as a solution to address these issues. These themes and sub-themes are shown in Table 2. S2 File illustrates representative quotations of all cohorts for each theme and sub-theme.

**Table 2:** Themes and sub-themes derived from thematic analysis of FGDs.

Themes	Sub-themes
**Theme 1:** Patient-Resident Gender Discordance	Hesitancy of Interacting with the Patient
	Deflection of Counseling the Patient
**Theme 2:** Ethnicity and Language Barriers	Patients Hailing from Different Ethnicities
	Inability to Translate Medical Terminologies to Urdu
**Theme 3:** Differing Social Class of the Patients	Socioeconomic Status of the Patient
	Education of the Patient
**Theme 4:** Challenging Patient-Resident Interactions	Patients Resistant to Treatment
	Multiple, Over-involved Attendants
	Inquisitive Patients and Those with Doctors in the Family
	Breaking Bad News and Complex Cases
**Theme 5:** Need for Developing a Curriculum Addressing Residents’ Communication Skills	Recommendations for Content
	Pedagogy and Delivery of the Curriculum

### Theme 1: Patient-resident gender discordance

When asked if patient gender influenced resident-patient communication, participants shared their observations of residents treating patients differently based on their gender. These included residents’ hesitancy in engaging in and deflection of counseling to the attendant in the case of physician-patient gender-discordant interactions.

#### Subtheme 1: Hesitancy of interacting with the patient

Participants reported that some female residents felt hesitant in talking to male patients. Male patients also felt more comfortable talking to male residents as compared to females:

*“Female residents feel a bit conscious around male patients, and vice versa, especially in general surgery clinics where the patient is quite vulnerable, and the exposure area is significant. I have seen such biases; they usually call in their male or female colleagues to come and attend to the patient….” (Fifth-year Medical Student, Female)*

#### Subtheme 2: Deflection of counseling the patient

Whilst residents commented that they provided the same treatment and counseling to patients regardless of their gender, it was observed by other healthcare providers (HCPs) that residents sometimes deflected their counseling based on the patient’s gender:

*“I’ve seen residents deflect their management or counseling to the attendant…For female patients, when residents see the male attendant, they start explaining the management to them [rather than the patient].” (Fifth-year Medical Student, 24 y Female)*

### Theme 2: Ethnicity and language barriers

Participants shared their observations of challenges faced by residents in providing culturally competent care to patients hailing from different ethnic backgrounds. These included language barriers and frequent instances of residents not being able to translate or explain things properly to patients in Urdu (the national language).

#### Subtheme 1: Patients hailing from different ethnicities

All cohorts agreed that there were frequent instances of language barriers between residents and patients owing to different ethnic backgrounds. While this hindered effective communication, residents appreciated the availability of language translators at the hospital. However, translators were not accessible all the time. In such cases, residents relied on ancillary staff and relatives of the patients to fill this gap. A senior resident reflected on how ethnic concordance between the patient and physician led to better communication and patient satisfaction:

*“I don’t know Sindhi, but I have colleagues who speak good Sindhi, so the satisfaction from the patient’s side is really great…if you know the patient’s language, he’s going to be very comfortable, and it’s going to be very easy to counsel.” (Fourth year surgical resident, 30 y Male)*

#### Subtheme 2: Inability to translate medical terminologies to Urdu

Faculty and fellows expressed concern that the existing training did not equip residents to translate medical terminologies into Urdu which caused a lag in effective patient-resident communication:

*“There is an overuse of medical jargon, be it residents, fellows or consultants because they are unaware of the Urdu terminologies. So, they need to be trained regarding the language that should be used during counseling.” (Medicine Fellow, 32 y Female)*

### Theme 3: Differing social class of the patients

In addition to the patients’ gender and ethnic background, social class of the patient was also highlighted by respondents as a factor influencing patient-resident communication. This included the patients’ socioeconomic status and level of education.

#### Subtheme 1: Socioeconomic status of the patient

Faculty and medical students commented that residents tended to treat patients with lower socioeconomic backgrounds differently than those from privileged backgrounds and form biases against patients, disregarding their economic limitations:

*“We think we can opt for any procedure to treat the patient, but we need to realize that a patient might be refusing treatment because they cannot afford it.” (Associate Professor, Obstetrics & Gynecology, 58 y Female)*

*“I have seen residents treat patients differently in the community health center clinics and executive clinics.” (Assistant Professor, Family Medicine, 50 y Female)*

On the contrary, residents denied having such biases, claiming that each patient was the same for them.

#### Subtheme 2: Education of the patient

It was also noticed that a difference in the education of the patient affected the degree of counseling by residents:

*“If there’s a more educated patient and it is understood that they expect a certain amount of understanding, more attention is given. With lower education from poorer backgrounds, there is a degree of dismissiveness. Residents just inform patients the date and time of their procedure rather than giving pertaining details.” (Fifth-year Medical Student, 24 y Female)*

### Theme 4: Challenging patient-resident interactions

Certain patient and attendant behaviors were cited as challenging for residents to deal with, highlighting the need for training in these areas. These included counseling patients who resist treatment, counseling multiple attendants of a single patient or those who asked a lot of questions and breaking bad news.

#### Subtheme 1: Patients resistant to treatment

Residents stated that it was difficult to counsel patients who were resistant to treatment, especially those who had searched up their symptoms online and developed a pre-conceived notion regarding a specific treatment.

*“More often than not, the first 2–4 hours are only taken in convincing the patient for a particular treatment and after that treatment starts…this is because Google is a patient’s best friend, and they have some preconceived treatment plan in mind.” (First Year Gynecology Resident, 26 y Female)*.

Residents often struggled while counseling non-compliant patients or probing them to understand their non-compliance. Hence, they relied on their seniors to perform this task. Dealing unsuccessfully with non-compliant patients also led residents to realize the gaps in their communication skills, for which they encouraged the idea of a communication skills curriculum.

#### Subtheme 2: Multiple, over-involved attendants

Patients with multiple attendants or those whose attendants had to be counseled over the phone were also challenging for residents:

*“Every attendant, maternal or paternal uncles, relatives other than the parents, want to talk to the doctors directly. It’s not possible to counsel every extended family member for a single patient.” (First Year Pediatrics Resident, 25 y Female)*

#### Subtheme 3: Inquisitive patients and those with doctors in the family

All cohorts believed that residents found it challenging to deal with patients who asked a lot of questions. Patients with doctors in their families were also reported by residents and faculty to be difficult to talk to since they cross-questioned treatment plans:

*“It depends on the attendant, if they are too fussy and their knowledge and education is a lot then they keep on asking you questions. (Pediatrics Fellow, 32 y Male)*

#### Subtheme 4: Breaking bad news and complex cases

According to fellows, the skill of breaking bad news was severely lacking in residents, irrespective of their training year. Even senior residents were not confident in delivering bad news independently. Hence, they relied on fellows for such counseling:

*“I have usually seen that residents tend not to do it and let the consultants do it because they’re very hesitant in talking about such news with the patient…they don’t know how the patients will react, or how much information to give at that moment. So, they let their superiors do it.” (Fifth Year Medical Student, 24 y Female)*

Regarding junior residents, fellows felt that they should be trained in delivering bad news early on in their residency but not break such news to patients independently owing to lack of adequate subject knowledge.

#### Theme 5: Need for developing a curriculum addressing residents’ communication skills

After identification of the existing problems in patient-resident communication, there was consensus regarding the need for a formal communication skills training curriculum for residents amongst all the cohorts. Participants gave their recommendations regarding the content of such a curriculum and the possible ways to deliver it.

#### Subtheme 1: Recommendations for content

Topics including breaking bad news, being empathetic to patients and learning Urdu translations of medical terminologies were the top suggestions to be included in the curriculum. A key subtheme that emerged was the importance of getting informed consent properly by patients undergoing interventional procedures. Surgical fellows noted that residents were not as careful as they should be:

*“I feel that sometimes while residents are taking the patient’s consent and making them sign the form, there are some minor flaws which can professionally create very big problems later. So, it is important to organize a communication skills program in which these things are standardized and taught to residents.” (Surgery Fellow, 36 y Male)*

#### Subtheme 2: Pedagogy and delivery of the curriculum

On the topic of utilizing different modalities for delivering a communication skills curriculum, workshops were recommended where residents could actively practice the skills learnt. Residents and fellows both opined that faculty should give feedback to residents after they counsel patients. Role-playing was another pedagogy highlighted for this purpose:

*“Role-playing will be effective in that [communication skills curriculum].” (First Year Gynecology Resident, 26 y Female)*

## Discussion

The FGDs conducted in this study with sixty HCPs demonstrated multifaceted patient factors and preconceived notions, including biases, amongst residents that hinder effective patient-physician communication. Thematic analysis revealed four major areas of concern preventing an open and constructive patient-resident dialogue, namely non-concordant patient-provider relationships, those with language barriers, patients belonging to diverse socioeconomic backgrounds and interaction of residents with difficult patients, who either resist treatment or are exceedingly inquisitive. Identification of these themes revealed a dire need for a formal and standardized communication skills curriculum for residents focusing on eliminating preconceived notions regarding patients while providing treatment.

Our study demonstrates that residents feel more confident in interacting with same-gender patients. Extensive literature suggests patients prefer same-gender physicians [17, 18]. Patient-doctor gender concordance has demonstrated higher satisfaction rates with both the communication process and treatment [19], while patient-physician gender discordance results in less trust [20].

Furthermore, differing cultural backgrounds add to these biases leading to inadequate patient-physician communication. Patient participation is a vital aspect of medical communication; however, it has been reported that patients from ethnic minorities contribute little to it [21]. Lack of cultural competency in resident-physicians translates into neglect towards and discrimination against such patients [21–23]. Additionally, use of medical jargon by residents worsens the impact of ineffective communication [24], as portrayed by our results.

All cohorts of HCPs in our study shared similar observations where they saw increased communication by residents with patients having a high social standing and education. Implicit bias in HCPs can negatively impact patient care, leading to the provision of poor-quality healthcare services. Even though these perceptions are unintentional, they can influence the patient-doctor relationship adversely. A systematic review showed discrepancies in doctors’ communication when dealing with patients from varying socioeconomic backgrounds [25]. Similar to this review, findings from another study indicated that doctors share more information with interactive patients that puts uninformed and uneducated patients from a lower social class at a disadvantage [26]. A study assessing unconscious social class bias in residents reported a strong preference towards upper class patients (Mean IAT D Score = 0.66) which might affect a patient’s quality of care, however it did not impact clinical decision-making [27].

In this age of enhanced information, it is easier for patients to search their symptoms online to reach a diagnosis and treatment plan, the reliability of which is often questionable [28]. Van Riel et al. reported that 45.71% of their study patients consulted the doctor after searching their health information online [29]. Doctor’s plans differing from internet search results often leads to patients questioning and arguing with the doctor, resisting their treatment or being non-compliant with follow-ups. As also stated by our study participants, this hampers physicians from counseling patients adequately and consumes more time [28]. Furthermore, our results indicated that residents feel overwhelmed while dealing with multiple attendants. Studies from Bahrain and Saudi Arabia, countries with similar cultural practices as Pakistan, reported HCPs to be reluctant in involving multiple family members in patient care, especially in settings requiring critical care or invasive interventions [30, 31]. However, residents’ perceptions in this aspect remained unexplored, making it a unique finding of our study. As reported in literature, our study also highlights hesitance of residents towards breaking bad news. A study in Pakistan reported 47% of residents being only fairly satisfied with their skills of breaking bad news [32], often due to their fears and limited time for protocol-oriented delivery of such sensitive information [33].

It is vital to incorporate a formal communication skills training curriculum within postgraduate medical education focusing on eliminating residents’ implicit biases towards patients and making them culturally competent. Literature shows lower confidence levels among residents when dealing with difficult patients or their attendants and delivering bad news [4, 34]. This is due to a dearth of training programs teaching residents how to cater to these situations efficiently. Similar to our study, integrating breaking bad news and the process of obtaining informed consent within these curricula have been highlighted by previous studies as well [4, 35]. Our study participants recommended videotaping, observation coupled with faculty feedback and role-plays for curriculum delivery. This is consistent with several studies favoring these methodologies [36], especially for teaching residents the skill of breaking bad news [37, 38].

Furthermore, identification of healthcare disparities has encouraged national initiatives in the United States, where funds are prioritized for research to alleviate these inequities [39]. Similarly, along with introducing a formal training curriculum, increased research opportunities can be made available in LMICs to highlight the gaps in patient-resident communication and provide solutions for it.

### Strengths and limitations

This study provides views from multiple perspectives regarding the impact of residents’ biases on their communication with patients leading to disparities in the care provided. In addition to residents self-evaluating themselves, this study also includes observations of faculty, fellows, and medical students—healthcare workers who work in close proximity with residents which has been suggested by prior studies [40]. Furthermore, congruency between our results and the literature adds strength to this study. This indicates a critical need for reducing the impact of patient disparities on residents’ communication. Whilst experiences from all these participants generated a holistic picture of patient-resident communication, this study is not without limitations. Firstly, we present perspectives from a single center which may hinder generalizability of findings. However, as this study was conducted at one of the largest AMCs, the patients and physicians represent diverse backgrounds and bring their distinctive characteristics within their consultations. Secondly, discussions with HCPs were conducted during COVID-19, where communication was severely impacted; use of personal protective equipment limited the practice of non-verbal communication skills, while the fear of contracting the virus decreased overall consultation time. These factors were not considered during data collection which might have biased residents’ perceptions during that time.

## Conclusion

Effective patient-resident communication is pivotal for providing high-quality healthcare. The diverse socioeconomic and cultural backgrounds of patients should not affect residents’ communication skills adversely. Adequate training of residents is required to improve their interpersonal and communication skills. Integrating strategies that can help residents in identifying and resolving their implicit biases towards patients can ensure empathetic delivery of care and improve patient outcomes.

## Supporting information

S1 File The consolidated criteria for reporting qualitative research (COREQ) checklist. (DOCX)

S2 File Representative quotations from qualitative focus group discussions. (DOCX)
