# Supplementary material for: Disparities in patient-resident physician communication and counseling: A multi-perspective exploratory qualitative study
Source: PLoS One. 2023 Oct 23;18(10):e0288549. doi: 10.1371/journal.pone.0288549 (PMC10593213; doi:10.1371/journal.pone.0288549)
Supplement: S2 File — (DOCX) [file pone.0288549.s002.docx]

**Table 3: Representative Quotations of Healthcare Providers Illustrating the Effect of Patient’s Socio-economic and Cultural Disparity on Their Communication**

| **Themes** | **Sub-themes** | **Representative Quotations** |
| --- | --- | --- |
| **Patient-Resident Gender Discordance** | Hesitancy of Interacting with the Patient | **Assistant Professor (Medicine), 40 y Male:** “Yes, I have seen residents treat certain types of patients differently based on the patients’ gender…”  **Fifth Year Medical Student, 24 y Female:** “Female residents feel a bit conscious around male patients, and vice versa, especially in general surgery clinics where the patient is quite vulnerable, and the exposure area is significant. I have seen such biases; they usually call in their male or female colleagues to come and attend to the patient.” |
|  | Deflection of Counseling the Patient | **Fourth Year Resident (Medicine), 30 y Female:** “I will talk to a male patient the same way I would talk to a female patient.”  **Fifth Year Medical Student, 24 y Female:** “I've seen residents actually deflect their management or counseling to the attendant…For female patients, residents turn around and when they see the male attendant, they start explaining the management to them [rather than the patient].” |
| **Ethnicity and Language Barriers** | Patients Hailing from Different Ethnicities | **Fellow (Medicine), 33 y Male:** “If it is the same language [between the patient and resident], there's more empathy naturally and more communication and the patient also wants to talk to the same resident because of ease of communication.”  **Fourth Year Resident (Surgery), 30 y Male:** “I don't know Sindhi, but I have colleagues that speak good Sindhi, so the satisfaction from the patient side is really great…if you know the patient's language, he's going to be very comfortable, and it's going to be very easy to counsel.” |
|  | Inability to Translate Medical Terminologies to Urdu | **Fellow (Medicine), 32 y Female:** “There is an overuse of medical jargon, be it residents, fellows or consultants because they are unaware of the Urdu terminologies. So, they need to be trained in this aspect regarding the language that should be used during counseling.”  **Second Year Resident (Surgery), 27 y Female:** “There are times where I have seen people talking to their patients in medical language or talking so fluently, that the patient is actually not able to understand what the doctor is trying to say…they are so complicated that the patient who is a lay man cannot understand it.” |
| **Differing Social Class of the Patients** | Socioeconomic Status of the Patient | **Associate Professor (Obstetrics & Gynecology), 58 y Female:** “We think we can opt for any procedure to treat the patient, but we need to realize that a patient might be refusing treatment because they cannot afford it.”  **Fourth Year Resident (Medicine), 30 y Female:** “As a doctor, I don’t differentiate between patients. For me, every socioeconomic class is the same…” |
|  | Education of the Patient | **Fellow (Surgery), 33 y Female:** “It depends more about how well-educated patients themselves are and that makes a difference on communication with the resident…”  **Fourth Year Medical Student, 23 y Female:** “: I have also seen this bias in residents. When they consider a patient to not be very well educated, they do not give them all the information.” |

**Table 4: Representative Quotations of Healthcare Providers Illustrating the Effect of Difficult Patients on their Communication**

| **Theme** | **Sub-themes** | **Representative Quotations** |
| --- | --- | --- |
| **Challenging Patient-Resident Interactions** | Patients Resistant to Treatment | **First Year Resident (Obstetrics & Gynecology), 26 y Female:** “There are some patients who are resistant to treatment from the very get go, and you form a preconceived notion about them…they are resistant to all your treatment plans. No matter how much we try to be non-judgmental, we do end up judging them.”  **First Year Resident (Pediatrics), 26 y Female:** “If a family, from the very beginning, shows such an attitude that they just don’t want a certain treatment, we don’t put that much effort in their counseling, not consciously though, I think we end up doing it unconsciously.”  **Fellow, (Obstetrics & Gynecology) 34 y Female:** “With non-compliant patients, they [residents] directly call consultants after they have tried their best to counsel the patient.”  **Fifth Year Medical Student, 24 y Female:** “There are very few residents who try to explore as to why a patient is not following medications or why are they being lost to follow up so there's very little effort into exploring why a patient is non-compliant and usually it automatically becomes the patient’s fault.” |
|  | Multiple, Over-involved Attendants | **Associate Professor, (Medicine), 50 y Male:** “The cultural context is very important. Over here, we cannot move ahead until and unless we have satisfied the attendants of the patient.”  **Fourth Year Resident (Medicine), 30 y Female:** “For every patient, we have 5-6 different attendants who we are asked to speak to. Some turn out to be very remote relatives, some even neighbors and they expect to know every single thing about the patient’s disease.” |
|  | Inquisitive Patients and Those with Doctors in the Family | **Fellow (Pediatrics), 32 y Male:** “It depends on the attendant, if they are too fussy and their knowledge and education is a lot then they keep on asking you questions.”  **First Year Resident (Obstetrics & Gynecology), 26 y Female:** “The other type of difficult patients are those who cross question you on your management because there is a doctor in their family or their medical knowledge is firm.” |
|  | Breaking Bad News and Complex Cases | **Fellow, (Medicine) 33 y Female:** “It’s easier to clutch onto your safety net and not take the responsibility on your shoulders. It’s easier to call the instructor at 2-3 am in the night and ask them to handle and troubleshoot for you instead of sorting things out on their own.”  **Fifth Year Medical Student, 24 y Female:** “I have usually seen that residents tend not to do it and let the consultants do it…they're very hesitant on talking about such news with the patient, because they don't know how the patients will react, or how much information to give to that patient at that moment. So, they let their superiors do it.” |

**Table 5: Representative Quotations of Healthcare Providers Illustrating the Need for Developing a Communication Curriculum for the Residents to Address Patient Biases During Their Dialogue and Counseling**

| **Theme** | **Sub-themes** | **Representative Quotations** |
| --- | --- | --- |
| **Need for Developing a Curriculum Addressing Residents’ Communication Skills** | Recommendations for Content | **Fellow (Surgery), 36 y Male:** “I feel that sometimes while residents are taking the patients’ consent and making them sign the form, there are some minor flaws [in counseling] which can professionally create very big problems later on. So over here it is important to organize a communication skills program or session in which these things are standardized and taught to the residents.”  **Second Year Resident (Surgery), 27 y Female:** “Communication with the patient, with the relatives, how to break bad news should be a priority for the curriculum, in addition to how to counsel for a simple procedure, about the consent etc.”  **Associate Professor, (Obstetrics & Gynecology) 58 y Female:** “We at the AMC think we can opt for any procedure to treat the patient but we need to realize that a patient might be refusing treatment because they cannot afford it. So, we need to teach this aspect to our trainees as well.” |
|  | Pedagogy and Delivery of the Curriculum | **Assistant Professor (Pediatrics), 55 y Female:** “Scripted recordings would be nice, like you know, for pediatrics or for medicine or surgery, and then letting them have a look at it and comment on maybe a perfect one and one with flaws so that they can compare both and say which one was a better one and why…”  **Fellow (Internal Medicine), 33y Male:** “One thing that can be done is the attendings and the supervisors with whom they [residents] do clinics on a daily basis, should ask the residents to counsel the patient [for observation and feedback].”  **Fourth Year Resident (Surgery), 30 y Male:** “So one component that can be added is that we are counseling the patient and our attending is observing us and then giving us feedback. So, at least in my department, this is lacking and this could really improve and expedite the learning curve.”  **First Year Resident (Obstetrics & Gynecology), 26 y Female:** “Role playing will be effective in that [communication skills curriculum].” |
